# The analysis of the genotype of Sapovirus outbreaks in Zhejiang Province

**DOI:** 10.1186/s12985-023-02202-z

**Published:** 2023-11-16

**Authors:** Lingxuan Su, Haiyan Mao, Yi Sun, Hao Yan, Qiong Ge, Liming Gong, Yanjun Zhang

**Affiliations:** 1 Zhejiang Provincial Center for Disease Control and Prevention, 3399 Binsheng Road, Hangzhou, 310051 China; 2 Key Laboratory of Public Health Detection and Etiological Research of Zhejiang Provincial, 3399 Binsheng Road, Hangzhou, 310051 China

**Keywords:** Sapovirus, Outbreak, Genotype, VP1 protein

## Abstract

**Background:**

Sapovirus (SaV) infection is increasing globally. Concurrently, several SaV-outbreaks were observed in children of Zhejiang province, China, in recent years, In this study, the genotypes of Sapovirus from seven outbreaks in the Zhejiang province were analysed.

**Methods:**

A total of 105 faecal samples were collected from children aged between 4 and 17 years from the Zhejiang Provincial Center for Disease Control and Prevention between October 2021 and February 2023. Genotypes were processed using reverse transcription polymerase chain reaction and Sanger sequencing, while next-generation sequencing was used to generate a complete viral genome. Deduced amino acid sequences were analysed to detect VP1 gene mutations.

**Results:**

In total, 60 SaV-positive patients were detected at a 57.14% (60/105) positivity rate. Positive rates in the seven outbreaks were: 22.22% (2/9), 15.00% (3/20), 93.10% (27/29), 84.21% (16/19), 28.57% (2/7), 53.33% (8/15) and 33.33% (2/6), respectively. Four genotypes were identified in the seven outbreaks, of which, GI.1 accounted for 14.29% (1/7), GI.2 accounted for 14.29% (1/7), GI.6 and GII.5 accounted for 14.29% (1/7), and GI.6 accounted for 57.14% (4/7). All patients were children and outbreaks predominantly occurred in primary schools and during cold seasons. Additionally, the complete sequence from the GI.6 outbreak strain showed high homology (identity: 99.99%) with few common substitutions (Y300S, N302S and L8M) in VP1 protein.

**Conclusions:**

SaV genotype diversity was observed in the seven outbreaks, with GI.6 being the main SaV genotype in Zhejiang province. It demonstrated high homology and may provide a platform for SaV prevention and control measures.

**Supplementary Information:**

The online version contains supplementary material available at 10.1186/s12985-023-02202-z.

## Introduction

Sapovirus (SaV) belongs to the *Sapovirus* genus of the *Caliciviridae* family and is a causative agent of acute gastroenteritis. SaV infects individuals of all age groups, and is implicated in both sporadic acute gastroenteritis cases and outbreaks [1]. SaV is also the main agent which causes acute gastroenteritis in children [2], with primary transmission via the faecal-oral route. Person-to-person transmission also occurs via faecal and vomit contact from SaV-positive patients or SaV-contaminated drinking water and food [3].

The SaV genome is a positive single-strand RNA, approximately 7.3–7.5 kb in size, and encodes two structural (VP1–VP2) and seven non-structural proteins (NS1–NS7). SaV is also highly diverse in terms of genes and antigens. The VP1 gene sequence is the main classification criterion-based on gene sequences, SaV is divided into 19 genogroups (GI–GXIX), with GI, GII, GIV, and GV primarily infecting humans, and the remaining genogroups primarily infecting animals [3]. The four human genogroups are further divided into 18 genotypes: GI.1–GI.7, GII.1–GII.8, GIV.1, GV.1 and GV.2, with GI.1 being the most globally prevalent genotype [2, 4]. VP1 is an essential structural protein for most capsid-related processes, such as assembly, host interactions and immunogenicity [5].

Acute gastroenteritis is a global public health issue, particularly in low- and middle-income countries [6]. Despite considerable vaccination efforts to reduce mortality, and other improved sanitation, water supply and public health awareness efforts, SaV infections remain a significant cause of death in young children [6]. Critically, in recent years, in rotavirus-mediated acute gastroenteritis, when a rotavirus vaccine program was implemented for children, morbidity, hospitalisation and mortality rates were dramatically reduced [7]. However, in a post-rotavirus vaccine era, other viral pathogens have been shown to cause acute gastroenteritis, e.g., SaV [8]. Globally, the positive rate of SaV in acute gastroenteritis ranged from 2.2 to 22.6%, and ranks second to fourth as a major viral pathogen causing disease [3, 9].

Since its first discovery in Japan in 1976, SaV sporadic cases and outbreaks in humans have been reported globally [10] in Sweden [11], The Netherlands [12], America [13] and Japan [14]. In 2010, Japan reported the largest foodborne SaV-outbreak involving 665 individuals [15]. Similarly, many provinces in China, including Yunnan [16], Guangdong [17, 18], Shandong [19] and Henan [20] have reported SaV epidemics. In 2014, a SaV GI.1 outbreak was reported in Zhejiang province [21], which hitherto had reported few outbreaks prior to this. Therefore, with potentially increasing SaV epidemics, SaV genotypes and VP1 protein mutations require comprehensive characterisation. To address this, seven SaV outbreaks were investigated to examine genotype characteristics and phylogenetic and amino acid mutations in the VP1 protein.

## Materials and methods

### Ethics statement

Research involving biological samples from humans was approved by the ethics committee of the Zhejiang Provincial Center for Disease Control and Prevention (Approval number: 2023-015-01).

### Patient inclusion and exclusion criteria

Refer to the Guidelines on Outbreak Investigation, Prevention and Control of Norovirus Infection (2015), within three days, the same gathering setting presented over five patients diagnosed acute gastroenteritis, with the epidemiological association, manifesting over three diarrhoea episodes or two vomiting episodes in 24 h, and at least two patients detected SaV positivity on laboratory diagnosis. Patients meeting these criteria were included. An epidemiological survey was performed by the Center for Disease Control and Prevention in Prefecture-level cities.

### Sample collection

In total, 105 faecal samples were collected from seven SaV acute gastroenteritis outbreaks (SX-2021-1, HuZ-2022-1, HuZ-2022-2, WZ-2022-3, JX-2022-4, TZ-2022-5, and JX-2023-1) from October 2021 to February 2023.

Faecal samples were prepared in 10% faecal phosphate-buffered saline (PBS) (Gibco, Bleiswijk, Netherlands), centrifuged at 4500 rpm at 4 °C for 30 min, and supernatants stored at − 80 °C. In nucleic acid assays, samples were negative for other viruses and bacteria associated with acute gastroenteritis (Norovirus, Astrovirus, Rotavirus, Adenovirus, Shigella, *Escherichia coli* and Salmonella).

### Viral nucleic acid extraction

Using a High Pure Viral Nucleic Acid Kit (Roche, Mannheim, Germany) and the manufacturer’s instructions, viral nucleic acid was extracted from 200 μL of faecal supernatant, eluted in 50 μL elution buffer, and stored at − 80 °C.

### SaV quantitative real-time reverse transcription polymerase chain reaction (qRT-PCR)

SaV qRT-PCR was conducted using the One Step PrimeScript™ RT-PCR Kit (TaKaRa, 064RA) in a final volume of 25 μL, containing 12.5 μL of buffer, 0.5 μL of *Taq*, 0.5 μL of RT enzyme, 0.6 μL of forward primer (20 μM), 0.6 μL of reverse primer (20 μM), 0.3 μL of probe (20 μM), 5 μL of template RNA, and 5 μL of RNase-free water. Amplification conditions were as follows: reverse transcription at 50 °C for 30 min, initial denaturation at 95 °C for 10 min, followed by 40 cycles of denaturation at 95 °C for 15 s, and annealing and extension steps at 62 °C for 1 min. Primer and probe sequences are listed (Additional File 1). qRT-PCR was performed on a 7500 Fast Real-time PCR System (Applied Biosystems).

### Reverse transcription PCR (RT-PCR) assay on partial VP1

A partial SaV VP1 (800 bp) sequence was amplified using the PrimeScript™ One-Step RT-PCR Kit Ver.2 (TaKaRa 055RA). The final reaction volume was 50 μL, containing 25 μL of buffer, 2 μL of enzyme mix, 1 μL of forward primer, 1 μL of reverse primer, 13 μL of RNase-free water, and 8 μL of RNA template. Forward primers were SVF13 and SVF14 and reverse primers were SVR13 and SVR14 as listed in Additional File 1. The RT-PCR amplification program of RT-PCR included: reverse transcription at 42 °C for 30 min and initial denaturation at 95 °C for 2 min, followed by 40 cycles of 95 °C for 30 s, 51 °C for 30 s, 72 °C for 1 min and 30 s, and a final extension at 72 °C for 10 min.

Nested RT-PCR was also performed to amplify the partial VP1 sequence [9] using the PrimeScript™ One-Step RT-PCR Kit Ver.2 (TaKaRa 055RA). First round PCR was performed in a final volume of 25 μL, containing 12.5 μL of buffer, 1 μL of enzyme mix, 2.5 μL of primer mix, 5 μL of RNase-free water, and 4 μL of RNA template. Forward primers were SaV124F, SaV1F and SaV5F, and reverse primers were SVR13 and SVR14 (Additional File 1). The first round amplification program was as described above. The second round was performed using TaKaRa *Taq*™ Hot Start Version (TaKaRa 007RA). The final volume was 50 μL and contained 5 μL of buffer, 4 μL of dNTPs, 0.25 μL of *Taq* enzyme, 0.5 μL of forward primer, 0.5 μL of reverse primer, 38.75 μL of RNase-free water, and 1 μL of first the PCR product. The forward primer was SV1245Rfwd and the reverse primer was SVR2 (Additional File 1). The amplification program was: initial denaturation at 94 °C for 2 min, followed by 35 cycles of 94 °C for 30 s, 50 °C for 30 s, 72 °C for 30 s, and a final extension at 72 °C for 5 min. Product sizes in first and second rounds were 800 bp and 430 bp, respectively. All products were sequenced using the Sanger sequencing method (YouKang Biological Technology).

### Next generation sequencing

A MATRIDX RT Kit (MATRIDX, Hangzhou, China) was used to synthesise double-stranded cDNA using random primers. cDNA was purified using Agencourt® AMPure® XP beads. Libraries were constructed using the MATRIDX Metagenomic DNA Library Construction Kit (MATRIDX, Hangzhou, China) and purified using Agencourt® AMPure® XP beads. The KAPA Library Quantification Kit for the Illumina® platform was used to determine library concentrations, after which, the library was sequenced using the Illumina NextSeq 2000 Reagent Kit P2 (100 cycles and single end) on an Illumina NextSeq 2000 platform.

Raw sequencing data were processed using quality control, alignment, and consensus sequence generation steps. Firstly, Fastq data were used to trim primer sequences and filter out low-quality reads. Secondly, all cleaned reads were mapped to the SaV reference sequence (Genbank: MN509083) using the Burrows-Wheeler Aligner. To eliminate duplicate sequences and non-unique mapped reads, used SAMtools to ulteriorly filter data. The sequencing depth was obtained using BCFtools. Finally, the remaining mapped reads were assembled to obtain whole sequences.

### Phylogenetic and amino acid analyses

DNA sequences were assembled using DNAMAN software, MEGA 7.0 was used to align sequences using the Clustal W method and generate a phylogenetic tree, which was constructed using the neighbour-joining algorithm and maximum composite likelihood model with 1000 bootstraps. The Geneious Prime (Ver. 2022.1.1) was used to align DNA and amino acid sequences using the MAFFT alignment method, and sites were confirmed according to the SaV strain (Hu/Nichinan-2-1-day3/2005/JP, GeneBank: AB455803). All reference sequences were obtained from the National Centre for Biotechnology Information (NCBI) GenBank database. All sequences obtained were submitted to GenBank (GenBank accession numbers: OR351085-OR351108).

## Results

### The epidemiological informations of seven outbreaks

In total, 105 cases form seven outbreaks were included; 9 patients from the SX-2021-1 outbreak, 20 from the HuZ-2022-1 outbreak, 29 from the HuZ-2022-2 outbreak, 19 from the WZ-2022-3 outbreak, 7 from the JX-2022-4 outbreak, 15 from the TZ-2022-5 outbreak, and 6 from the JX-2023-1 outbreak. All outbreaks were well distributed across the province; SX-2021-1, HuZ-2022-1, HuZ-2022-2, JX-2022-4 and JX-2023-1 occurred in the north of Zhejiang province, while TZ-2022-5 and WZ-2022-3 occurred in the midlands and south, respectively. Patients in outbreaks manifested with mild stomachache, diarrhoea, vomit, nausea and fever symptoms. All outbreaks lasted for 3–5 days.

As indicated (Table 1), 60 SaV-positive patients were detected with a 57.14% positive rate (60/105). Positive rates in SX-2021-1, HuZ-2022-1, HuZ-2022-2, WZ-2022-3, JX-2022-4, TZ-2022-5 and JX-2023-1 outbreaks were: 22.22% (2/9), 15.00% (3/20), 93.10% (27/29), 84.21% (16/19), 28.57% (2/7), 53.33% (8/15) and 33.33% (2/6), respectively.

**Table 1:** Distribution of seven outbreaks of SaV according to gender, age, month and genotypes Epidemiological information on seven outbreaks in Zhejiang province, China

Cases	Total cases	Positive cases, n (%)	Gender, n (%)		Median age	Settings	Genotype	Month
			Male	Female				
SX-2021-1	9	2 (22.22)	5 (55.56)	4 (44.44)	7	Primary school	GI.1	October
HuZ-2022-1	20	3 (15.00)	9 (45.00)	11 (55.00)	5	Kindergarten	GI.6	November
HuZ-2022-2	29	27 (93.10)	13 (44.83)	16 (55.17)	11	Primary school	GI.6 + GII.5	November
WZ-2022-3	19	16 (84.21)	12 (63.16)	7 (36.84)	16	Senior high school	GI.2	February
JX-2022-4	7	2 (28.57)	1 (14.29)	6 (85.71)	5	Kindergarten	GI.6	October
TZ-2022-5	15	8 (53.33)	6 (40.00)	9 (60.00)	10	Primary school	GI.6	November
JX-2023-1	6	2 (33.33)	3 (50.00)	3 (50.00)	10	Primary school	GI.6	February
Total	105	60 (57.14)	49 (46.67)	56 (53.33)	10	/	/	/

### Age and setting characteristics

Median patient ages across outbreaks (SX-2021-1, HuZ-2022-1, HuZ-2022-2, WZ-2022-3, JX-2022-4, TZ-2022-5 and JX-2023-1) were 7, 5, 11, 16, 5, 10 and 10 years old, respectively, and all were children < 18 years old (Table 1). Additionally, all outbreaks occurred in schools, including kindergartens (2/7, 28.57%), primary schools (4/7, 57.14%) and senior high schools (1/7, 14.29%). Therefore, primary schools were primary outbreak locations.

### The season of SaV outbreaks

As indicated (Table 1), two outbreaks (WZ-2022-3 and JX-2023-1) occurred in February, two (SX-2021-2 and JX-2022-4) in October and three (HuZ-2022-1, HuZ-2022-2 and TZ-2022-5) in November. Occurrence times (months) were based on seasons of China, with outbreaks occurring in cold season. Therefore, in China, SaV appeared to circulate more in cold seasons in our cohort.

### The genotypes of SaV outbreaks

**Fig. 1:**
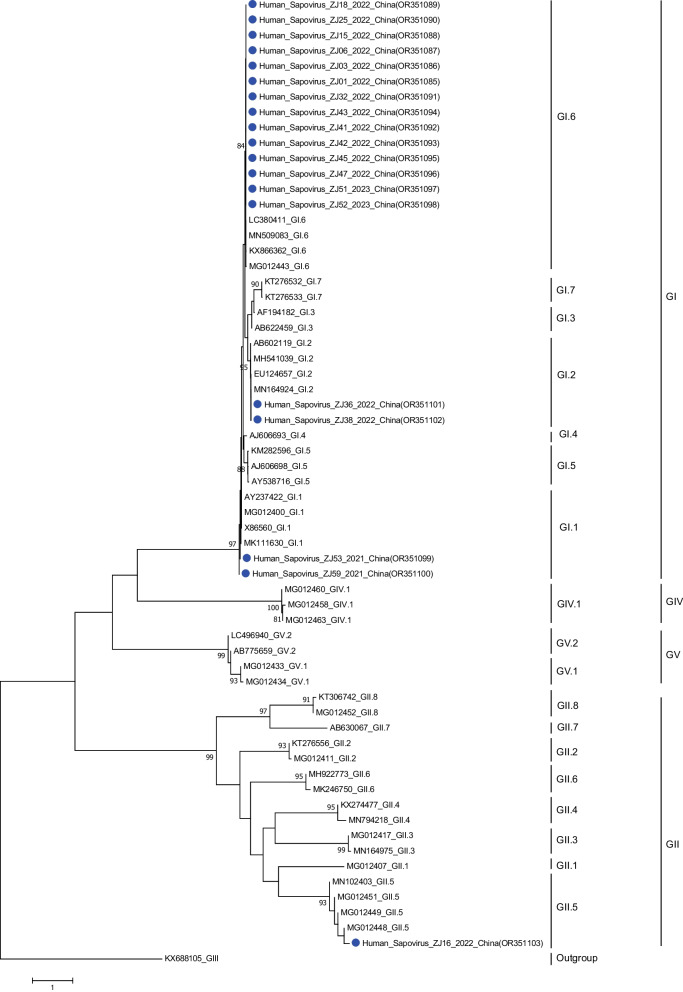
Phylogenetic analysis of SaV *VP1* gene nucleotide sequences. A phylogenetic tree was constructed using neighbour-joining algorithms. Blue circles indicate Zhejiang province SaV strains

**Fig. 2:**
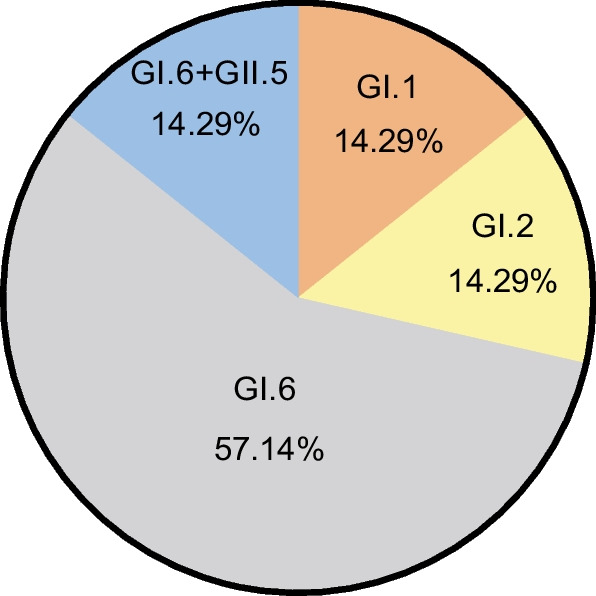
SaV outbreak genotypes

Partial VP1 was amplified from samples at partial lengths of 800 or 430 bp; 38 partial VP1 sequences successfully generated. From phylogenetic tree analyses (Fig. 1), the SX-2021-1 outbreak was GI.1, WZ-2022-3 was GI.2, and HuZ-2022-1, JX-2022-4, TZ-2022-5 and JX-2023-1 outbreaks were GI.6, while the HuZ-2022-2 outbreak was GI.6 and GII.5 combined. As shown (Fig. 2), GI.1 accounted for 14.29% (1/7), GI.2 accounted for 14.29% (1/7), GI.6 and GII.5 accounted for 14.29% (1/7) and GI.6 accounted for 57.14% (4/7). Additionally, the combined GI.6 and GII.5 outbreak yielded 18 partial *VP1* sequences, including 17 of GI.6 sequences and 1 of GII.5 sequence.

From phylogenetic tree analyses, GI.1, GI.2 and GI.6 sequences were located on the same evolutionary gradient, therefore, they were homologous but belonged to different outbreaks. Based on these data, SaV genotype diversity had occurred in outbreaks, but GI.6 was dominant across the seven outbreaks, particularly in 2022.

### VP1 amino acid analysis

GI.6 was dominant across the seven outbreaks, with five complete sequences generated. According to GI.6 whole nucleic acid sequence alignments, protein identity was 99.99% and indicated high homology. Therefore, the GI.6 SaV outbreaks demonstrated high homology, and suggested that outbreaks were possibly generated by the same strain. Additionally, when compared with whole SaV genomes from Japan (Sapovirus/Hu/GI.6/Nichinan/FP05284/2005/JPN, Genbank: LC380411) and the USA (Hu/US/2015/GI.6/Nashville9367, Genbank: MG012443; Hu/US/2016/GI.6/CA-RGPS-1084, Genbank: MN509083) in NCBI, SaV strain identities were 97.8% (Japan-LC380411), 97.8% (USA-MG012443) and 97.8% (USA-MN509083). Thus, all strains demonstrated high homology.

**Fig. 3:**
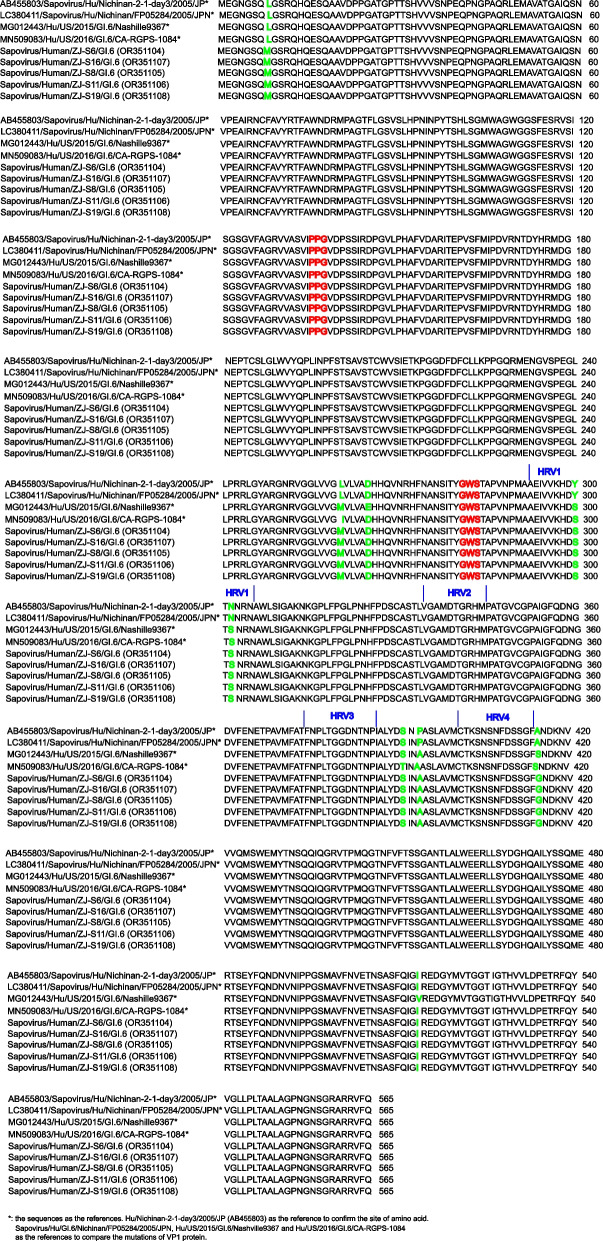
GI.6 VP1 amino acid sequence

Amino acid sequences of the complete VP1 region of GI.6 were analysed (Fig. 3). Complete *VP1* measured 1698 bp and contained 565 amino acids. Hu/Nichinan-2–1-day3/2005/JP as the reference to confirm the site of amino acid. Sapovirus/Hu/GI.6/Nichinan/FP05284/2005/JPN, Hu/US/2015/GI.6/Nashville9367 and Hu/US/2016/GI.6/CA-RGPS-1084 as the references to compare the mutations of VP1 protein. Four highly variable regions (HVR1, HVR2, HVR3 and HVR4) were identified in P2 of VP1. Amino acid sequence comparison analyses of these four regions showed that the GI.6 sequence contained two amino acid substitutions (Y300S and N302S) in HVR1. One amino acid substitution (A415G) in HVR4 compared with Sapovirus/Hu/GI.6/Nichinan/FP05284/2005/JPN, but when compared with the other two strains (Hu/US/2015/GI.6/Nashville9367 and Hu/US/2016/GI.6/CA-RGPS-1084), HVR4 exhibited an amino acid substitution (S415G). Additionally, it exhibited other amino acid mutations in VP1. When compared with Sapovirus/Hu/GI.6/Nichinan/FP05284/2005/JPN, three amino acid mutations (L8M, L260M and P395A) were identified. When compared with Hu/US/2015/GI.6/Nashville9367, three amino acid mutations (L8M, E265D and V514I) were identified. When compared with Hu/US/2016/GI.6/CA-RGPS-1084, three amino acid mutations (L8M, I260M and P392A) were identified.

## Discussion

Zhejiang province is located in southeast China, covers 105,500 square kilometres and contains 11 cities. Approximately 65.77 million people live in the province, of which 9.07 million are children. The province is economically prosperous with considerable transportation hubs and good internal and external communication links. Thus, viral transfer is easily facilitated. SaV is an important virus as it causes acute gastroenteritis in children, often causing outbreaks and leading to public health issues. To understand more about the seven recent SaV outbreaks in Zhejiang province, we investigated these outbreaks in depth, reported genotypes, and identified VP1 amino acid mutations.

SaV mainly infects individuals with low immunity, such as children and the elderly, but children under 5 years old are more often targeted [22] as indicated in a previous study where SaV was dominant in infants under 24 months old [23]. Moreover, elderly individuals over 60 years old are also at risk [12]. However, from our study, children over 5 years old were predominantly infected, which contrasted with previous studies. Therefore, acute gastroenteritis caused by SaV in children over 5 years old cannot be ignored. SaV infections exhibit no seasonal characteristics and may occur throughout the year [22], however, cold seasons (Autumn and Winter) are the main SaV infectivity periods [3, 24]. This was consistent with our data where the seven outbreaks occurred in cold months (October, November and February).

SaV is genotypically diverse, however, GI and GII genogroups are globally dominant [25, 26]. From several studies, the GI.1 genotype is the most common. In Japan, GI.1 and GI.2 are the main epidemic genotypes, with GI.1 having the dominant position [27–29]. In Thailand, GI.1 detection rates, followed by GI.2 and GII.5, were highest between 2019 and 2020 [30]. In Canada, the main SaV epidemic genotypes are GI.1, GI.2 and GII.5 [31], while in China, GI.1 is the most common. A previous 2-year study in Chongqing reported that GI.1 and GI.2 were the most prevalent genotypes [32], while in Guangzhou, SaV genotyping in patients with acute gastroenteritis showed that GI.1 was the most common genotype, followed by GI.2 [17]. However, a study from the Western Amazon reported that GIV.1 was the most prevalent genotype [33].

In our study, GI.1 and GI.2 were detected, but GI.6 was dominant across the seven outbreaks. GI.6 was also the main genotype in the SaV outbreak in Osaka, Japan, from 2004 to 2005 [34]. However, in Taiwan, GI.1, GII.3 and GII.8 were identified in outbreaks between 2012 and 2014 [35]. In Shenzhen, China, GI.2, GII.3 and GII.8 were also identified in outbreaks [18, 36]. Globally, GI.6 was detected in patients and outbreaks, but at low detection rates in Brazil [37], Germany [38], Thailand [39], America [10] and Japan [40]. Moreover, GI.6 was the main genotype in Tibet [41]. Additionally, South Africa [42], Brazil [43], Japan [44], Italy [45] and Shandong province, China [9] identified GI.6 in sewage, which suggested this genotype may be prevalent on a large scale. Additionally, GII.5 is a rare genotype and has low global detection rates in Africa, Taiwan and other regions [26, 35].

The VP1 protein is one of the main structural proteins in the SaV virion and *VP1* gene variability is the main SaV classification criterion [3]. As viruses cannot be cultivated, receptor interactions with hosts remain unclear. Previous studies have reported that the two highly conserved motifs, "PPG" and "GWS" in the VP1 region help maintain viral structural stability [5]. In our study, no mutations were identified at "PPG" and "GWS" in the GI.6 strain. Additionally, four highly variable regions in VP1 (HVR1–HVR4) may have key roles in SaV antigenic diversity and immunogenicity [5]. Two amino acid substitutions were identified in HVR1 (Y300S and N302S) and one substitution in HVR4 (A415G to S415G) in GI.6. However, it remains unclear if variations in these amino acids affect SaV antigenicity and immunogenicity.

## Conclusions

GI.6 was the dominant genotype in seven SaV outbreaks in Zhejiang province, China. Highly homology and genotype diversity was also identified in outbreaks. Children and schools were the primary targets. Therefore, more studies and surveillance programs are required to investigate the molecular epidemiology underpinning human SaV.

### Supplementary Information

**Additional file 1**. Primer sequences used in the study

## Data Availability

Datasets and analyses used in this study are available from the corresponding author upon reasonable request

## Abbreviations

SaV: Sapovirus
PBS: Phosphate-buffered saline
NCBI: National Centre for Biotechnology Information
RNA: Ribonucleic acid
RT-PCR: Reverse transcription polymerase chain reaction
qRT-PCR: Quantitative real-time reverse transcription polymerase chain reaction
