# Supplementary material for: The analysis of the genotype of Sapovirus outbreaks in Zhejiang Province
Source: Virol J. 2023 Nov 16;20:268. doi: 10.1186/s12985-023-02202-z (PMC10655437; doi:10.1186/s12985-023-02202-z)
Supplement: Supplementary file 1 — Additional file 1. Primer sequences used in the study [file 12985_2023_2202_MOESM1_ESM.docx]

|  | primers | Sequences (5’→3’) |
| --- | --- | --- |
| Primers of VP1 amplification  Primers of qRT-PCR | SaV124F  SaV1F  SaV5F  SVF13  SVF14  SVR13  SVR14  SV1245Rfwd  SVR2  SaV124F  SaV1F  SaV5F  SaV1245R  SaV124TP  SaV5TP | GAYCASGCTCTCGCYACCTAC  TTGGCCCTCGCCACCTA  TTTGAACAAGCTGTGG  GAYYWGGCYCTCGCYACCTAC  GAACAAGCTGTGGCATGCTAC  GGTGANAYNCCATTKTC  GGTGAGMMYCCATTCC  TAGTGTTTGARATGGAG  GWGGGRTCAACMCCW  GAYCASGCTCTCGCYACCTAC  TTGGCCCTCGCCACCTAC  TTTGAACAAGCTGTGGCATGCTAC  CCCTCCATYTCAAACACTA  FAM-CCRCCTATRAACCA-MGB-NQF  FAM-TGCCACCAATGTACCA-MGB-NQF |

Additional file 1 Primer sequences used in the study
